# Characterization of teaching-learning tutors in public health services and topics of interest for their work

**DOI:** 10.1590/1980-220X-REEUSP-2023-0268en

**Published:** 2024-03-29

**Authors:** Isadora Siqueira de Souza, Francisco Timbó de Paiva, Rebecca Cabral de Figueirêdo Gomes Pereira, Guilherme Barbosa Shimocomaqui, Ana Cláudia Pereira da Paz, Marcio Anderson Cardozo Paresque, Ilana Eshriqui

**Affiliations:** 1 Hospital Israelita Albert Einstein, Centro de Estudos Pesquisa e Prática em Atenção Primária à Saúde e Redes, São Paulo, SP, Brazil.

**Keywords:** Mentoring, Public Health Services, Education, Continuing, Planning, Tutoría, Servicios Públicos de Salud, Educación Continua, Planificación, Tutoria, Serviços Públicos de Saúde, Educação Continuada, Planejamento

## Abstract

**Objective:**

To describe the profile of teaching-learning tutors in public health services and investigate which topics are of greatest interest in development spaces for these actors.

**Method:**

Cross-sectional study. Eligible tutors of Health Care Planning. Data collection using an electronic questionnaire composed of closed questions on sociodemographic characteristics, training and performance. Chi-square test used to compare proportions according to tutor typologies.

**Results:**

A total of 614 tutors worked in Brazil’s five geographic regions, the majority in primary care (82%), followed by state/regional work (13%) and specialized outpatient care (5%). The majority reported being female, of brown skin color, from the nursing field, having worked as a tutor for less than a year, and with no previous experience in preceptorship or similar. The most important topics were Health Care Networks, risk stratification for chronic conditions and the functions of specialized outpatient care.

**Conclusion:**

The predominance of certain characteristics among tutors was identified, with differences between the types of work. The findings can support managers in the process of selecting and developing tutors in Health Care Planning.

## RESUMO

**Objetivo:**

Descrever o perfil de tutores de ensino-aprendizagem em serviços públicos de saúde e investigar quais são os temas de maior interesse em espaços de desenvolvimento desses atores.

**Método:**

Estudo transversal. Elegíveis tutores da Planificação da Atenção à Saúde. Coleta de dados por questionário eletrônico composto por questões fechadas sobre características sociodemográficas, formação e atuação. Teste Qui-quadrado utilizado para comparar proporções segundo tipologias de tutor.

**Resultados:**

Considerados 614 tutores, que atuavam nas cinco regiões geográficas brasileiras, sendo a maioria na Atenção Primária (82%), seguido por atuação estadual/regional (13%) e na Atenção Ambulatorial Especializada (5%). A maioria referiu ser mulher, de cor da pele parda, da área de enfermagem, atuação como tutor há menos de 1 ano, e sem experiência prévia em preceptoria ou similar. Temas considerados mais importantes destacam-se Redes de Atenção à Saúde, estratificação de risco de condições crônicas e funções da Atenção Ambulatorial Especializada.

**Conclusão:**

Identificou-se a predominância de algumas características entre tutores, com diferenças entre as tipologias de atuação. Os achados podem apoiar gestores no processo de seleção e desenvolvimento de tutores na Planificação.

## RESUMEN

**Objetivo:**

Describir el perfil de los tutores de enseñanza-aprendizaje de los servicios de salud pública e investigar los temas de mayor interés en espacios de desarrollo de estos actores.

**Método:**

Se trata de un estudio transversal con Tutores Elegibles de Planificación de la atención en salud; con datos recopilados mediante cuestionario electrónico de preguntas cerradas sobre características sociodemográficas, formación y actuación. Se utilizó la prueba de Chi-cuadrado para comparar las proporciones según las tipologías del tutor.

**Resultados:**

Se consideraron 614 tutores que trabajaban en las cinco regiones geográficas del Brasil, la mayoría en Atención Primaria (82%), seguido de actuación estatal/regional (13%) y Atención Ambulatoria Especializada (5%). La mayoría declaró ser mujer, de piel morena, del ámbito de la enfermería, con menos de un año de actuación como tutora y sin experiencia previa como preceptora o similar. Los temas más importantes fueron las Redes de la Atención en Salud, la estratificación del riesgo de las enfermedades crónicas y el papel de la Atención Ambulatoria Especializada.

**Conclusión:**

Se identificó el predominio de ciertas características entre los tutores, distintas según los tipos de actuación. Las conclusiones pueden servir de apoyo a los gestores en el proceso de selección y desarrollo de los tutores en Planificación.

## INTRODUCTION

Permanent Health Education (PHE) is a competence of the Unified Health System (SUS) and has been the target of various Brazilian public actions and policies over the last three decades^(1)^. The National Policy for Permanent Education in Health stands out, as its fundamental principle is to improve the qualifications of health professionals. In this context, PHE is a tool that aims to transform practices in health services daily. It should be understood as a teaching-learning practice and also as an education policy in the area of health, the aim of which is to generate knowledge based on the daily experience of health institutions, taking into account the reality experienced by the players involved^(2)^.

Since the 8^th^ National Health Conference and the 1^st^ National Conference on Human Resources in Health^(3)^, The training of health workers for the SUS is a major challenge. We are looking for initiatives that respond to the health needs of the Brazilian population, that understand the social determination of the health-disease process, the importance of the Health Care Network (HCN) and the integration of Primary Health Care (PHC) points with Specialized Outpatient Care (SOC)^(4)^. In this sense, the National Specialized Care Policy, established in 2023^(5)^, points out that specialized service teams should develop strategies for permanent education, support and shared care, providing support for PHC reference teams.

The need for constant improvement of health services and professionals is exacerbated as technological advances accelerate. In developing countries with continental dimensions, such as Brazil^(6)^, there is a gap between the most modern technologies applied to health and the social and health reality of a large part of the population, who live in conditions of extreme deprivation. To reduce the impact of these shortcomings, work-based learning programs have been designed to produce and systematize knowledge related to the development of health work^(1,7)^.

Historically, experiences such as the Pan American Health Organization’s (PAHO) *Projeto Larga Escala* (PLE) have contributed to establishing a context that would enable the transformation of health services, whose conceptual basis required the construction of methodological teaching alternatives^(8)^. Considering the population subjected to the risks of care provided by unqualified workers, the PLE was committed to reorganizing health services based on the qualification of professionals^(9)^. One of PLE’s inspirations was the French movement Peuple et culture (People and Culture), which, since 1945, has developed popular education approaches, promoting critical education as a large-scale education strategy through educators/mediators^(10)^.

In Brazil, in 1985, the foundation of the Joaquim Venâncio Polytechnic Health School of the Oswaldo Cruz Foundation (Fiocruz) already represented an PHE initiative, through a partnership between the education and health spheres, with a view to articulating different areas of professional health training^(11)^. From the outset, its actions were geared towards training that would enable students to acquire technical and operational knowledge and the scientific and philosophical foundations that guide the respective types of work. The Health Work Education Program (PET-Health Program) has been an initiative of the Brazilian government since 2008, with the aim of integrating teaching, service and the community. Its aim is to qualify the academic professional training and continuing education of SUS and university professionals for comprehensive health care.

Another initiative developed in Brazil refers to Health Care Planning (HCP), proposed in 2004 as a methodology that aims to support the technical and managerial staff of state and municipal health departments in organizing work processes in the context of the HCN. In this sense, HCP proposes the development of health team competencies for the organization of Health Care, focusing on the needs of the users under their responsibility^(12)^. Among other practices, HCP consists of management consultancy, the tutoring process in health services and short-term training for care professionals and managers, aimed at organizing the work process.

These movements have a common question: “How can we provide workers with the skills and knowledge to think and act through educational processes linked to services?” In this context, we are interested in further investigating the profile and understanding of the tutors involved in the educational and operational processes in a HCP project. Considering that this role is under constant construction and that, currently, the profile of professionals who deal with PHE processes from the perspective of tutoring in the HCP is not established in the literature, this article aims to describe the profile of tutors who work in teaching-learning actions in health services that develop the HCP methodology, as well as to investigate which are the topics of greatest interest to be worked on in development spaces for these actors.

## METHOD

### Type

This is a cross-sectional, observational, descriptive study with a quantitative approach, guided by the STrengthening the Reporting of OBservational studies in Epidemiology (STROBE) checklist^(13)^, which contains 22 verification items, with recommendations for a complete and accurate description of observational studies.

### Setting

The study was carried out in the context of the Program of Support for the Institutional Development of the Unified Health System (PROADI-SUS) project entitled “The organization of specialized outpatient care in a network with primary health care”, known as *PlanificaSUS*, which has been running since 2018, with the aim of supporting the implementation of the Health Care Planning (HCP) methodology^(14)^. Participating in *PlanificaSUS* are 24 health regions distributed across Brazil’s five geographic regions: 5 in the North, 9 in the Northeast, 4 in the Midwest, 3 in the Southeast and 3 in the South. In each of the health regions, a Specialized Outpatient Care (SOC) service and Primary Health Care (PHC) services are listed for implementation of the HCP methodology.

### Population and Selection Criteria

The study population was made up of health professionals who work as *PlanificaSUS* tutors. The third generation of the HCP methodology works with the improvement model and the concept of tutorial education, i.e. everyone teaches and everyone learns^(12)^. The tutor is set up as the central figure for monitoring the set of process changes in the services and has mastery of the how-to.

**Figure 1:**
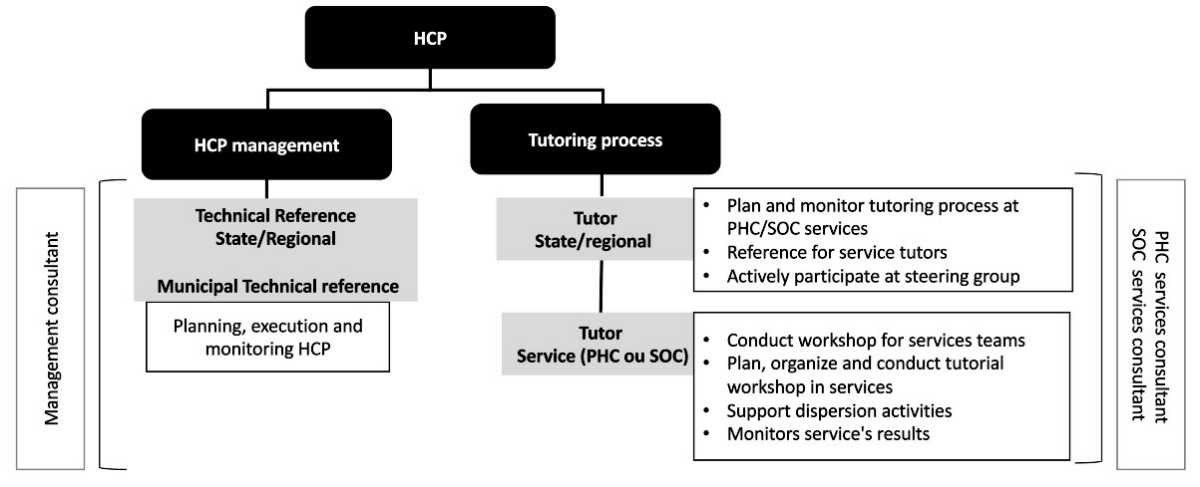
Description of the roles in Health Care Planning.

Note: HCP (Health Care Planning); PHC (Primary Health Care); SOC (Specialized Outpatient Care).

Figure 1 shows the division of the leading roles in the implementation of the HCP, highlighting, for this study, the tutors, who are responsible for the tutoring process in their respective health regions, municipalities or health units. From this perspective, the tutors at state and regional level represent references for the development and performance of the service tutors and for discussing the topics about the tutoring processes with management. The PHC and SOC services tutors interact directly with the professionals, carrying out educational work and intervening in reality.

The exclusion criteria were not being registered on the e-Planifica platform, used to manage and monitor *PlanificaSUS* ^(14)^ (https://planificasus.com.br/) (n = 73) or not indicating that they were a tutor in at least one of the defined typologies (state, regional, service of PHC and/or SOC) (n = 82).

This study opted for sampling based on the “snowball” method, carried out by sending invitations by email and WhatsApp groups to the actors involved in PlanificaSUS. This type of method, also known as the chain of references method, begins with the inclusion of a certain number of people who are part of the target population, who can indicate from their contacts other individuals for the sample. This continues successively until the desired number is reached^(15)^. Thus, the final sample consisted of 614 participants.

### Data Collection

Data was collected between August and September 2022, using a structured questionnaire prepared by the authors, which consisted of closed questions, including sociodemographic characteristics (gender, age group, race/color, schooling, geographic region) and those related to training and work in the HCP (degree area, highest degree of training, type of tutor, time since graduation, time working as a tutor, protected weekly hours, working as a tutor in the same workplace, previous work as a preceptor or similar, perception of important themes to be developed and time of experience in PHC and in SOC units in the Outpatient Secondary Care Point model (from Portuguese, Ponto de Atenção Secundária Ambuatorial – PASA). The questionnaire was prepared in electronic format and made available using the Research Electronic Data Capture (REDCap©) software^(16)^, upon presentation and acceptance of the Informed Consent Form (ICF).

### Data Analysis and Treatment

Categorical variables were described using frequency and proportions. The Chi-square test was used to compare the proportions of the descriptive variables according to the types of tutor (state/regional, PHC service, SOC service). The continuous variable age was described using the mean and standard deviation. The bar graph was drawn up to show the percentage of responses per category of type of tutor, on the topics identified as important to be developed in order to work as a tutor. Stata software version 13.0 was used for the statistical analysis.

### Ethical Aspects

The study was approved by the Research Ethics Committee of Hospital Israelita Albert Einstein, under protocol CEP 3.674.106 in 2019, in accordance with Resolution No. 466/2012 of the National Health Council. Participation took place upon presentation and acceptance of the ICF.

## RESULTS

A total of 614 *PlanificaSUS* tutors took part in the study, with a mean age of 36.8 years (21.8 – 71.5, SD = 8.8 years). Table 1 shows the general characterization of the participating tutors, the majority of whom identified themselves as cisgender women (87%), with a degree in nursing (81%) and white or brown race/color (90%), with around 10% of the participants identifying themselves as black, yellow or indigenous.

**Table 1:** Sociodemographic and training characteristics of *PlanificaSUS* tutors – São Paulo, SP, Brazil, August-September, 2022.

Characteristics	N (%)
**Gender (n = 609)**	
Cisgender woman	530 (87.0)
Cisgender man	71 (11.7)
Transsexual or non-binary	8 (1.3)
**Race / color (n = 611)**	
White	243 (39.8)
Brown	306 (50.1)
Black	43 (7.0)
Yellow	7 (1.1)
Indigenous	12 (2.0)
**Geographical region (n = 605)**	
North	116 (19.2)
Northeast	265 (43.8)
Midwest	52 (8.6)
Southeast	63 (10.4)
South	109 (18.0)
**Degree (n = 551)**	
Nursing	448 (81.3)
Psychology	10 (1.8)
Dentistry	21 (3.8)
Nutrition	14 (2.5)
Social Work	16 (2.9)
Physiotherapist	10 (1.8)
Others	32 (5.8)
**Type of tutor (n = 614)**	
State or Regional	77 (12.6)
PHC Service	505 (82.2)
SOC Service	32 (5.2)

Note: PHC (Primary Health Care); SOC (Specialized Outpatient Care).

The participants lived in the five geographical regions of Brazil, with the majority in the Northeast (43.8%), followed by the North (19.2%), South (18.0%), Southeast (10.4%) and Midwest (8.6%). As for the type of tutor, the majority worked in PHC services (82.2%), followed by around 12% who worked at state or regional level, and 5% in SOC services (Table 1). Table 2 shows the training and work characteristics of the different types of tutors.

**Table 2:** Characteristics related to the performance of tutors in *PlanificaSUS*, by type of performance – São Paulo, SP, Brazil, August-September, 2022.

Characteristics	Total n (%)	Type of tutor n(%)			P-value
		State or regional	PHC service	SOC service	
**Length of time as a tutor**					
<1 year	358 (58.3)	37 (48.5)	306 (60.6)	15 (46.9)	**0.012**
Between 1 and 2 years	144 (23.4)	16 (20.8)	121 (24.0)	7 (21.9)	
>2 years and <5 years	106 (17.3)	23 (30.0)	73 (14.5)	10 (31.2)	
≥5 years	6 (1.0)	1 (11.3)	5 (1.0)	0 (0)	
**Time since graduation**					
≤1 year	30 (4.9)	5 (6.5)	23 (4.6)	2 (6.2)	**<0.001**
>1 and ≤5 years	177 (29.1)	12 (15.6)	163 (32.6)	2 (6.2)	
>5 and <10 years	125 (20.5)	9 (11.7)	110 (22.0)	6 (18.8)	
≥10 years	277 (45.5)	51 (66.2)	204 (40.8)	22 (68.8)	
**Higher level of education**					
Technical or secondary education	6 (1.0)	1 (1.3)	4 (0.8)	1 (3.1)	**<0.001**
Full degree	213 (34.9)	9 (11.7)	198 (39.5)	6 (18.7)	
Residency	13 (2.1)	2 (2.6)	8 (1.6)	3 (9.4)	
Lato-Sensu Specialization	343 (56.2)	53 (68.8)	270 (53.9)	20 (62.5)	
Stricto-Sensu Post-Graduation	35 (5.7)	12 (15.6)	21 (4.2)	2 (6.3)	
**Protected weekly hours**					
None	264 (43.8)	24 (32.0)	227 (45.8)	13 (40.6)	**0.004**
Up to 2 hours	172 (28.5)	26 (34.7)	141 (28.4)	5 (15.6)	
More than 2 hours	167 (27.7)	25 (33.3)	128 (25.8)	14 (43.8)	
**Acting as a tutor in the same workplace**					
No	122 (20.2)	34 (44.7)	82 (16.6)	6 (18.7)	**<0.001**
Yes	481 (79.8)	42 (55.3)	413 (83.4)	26 (81.3)	
**Previous experience as a preceptor**					
No	365 (59.6)	40 (51.9)	308 (61.1)	17 (54.8)	0.267
Yes	247 (40.4)	37 (48.1)	196 (38.9)	14 (45.2)	
**PHC experience**					
No experience	67 (10.9)	18 (23.4)	38 (7.5)	11 (34.4)	**<0.001**
Up to 1 year	79 (12.9)	8 (10.4)	68 (13.5)	3 (9.4)	
>1 and ≤5 years	220 (35.9)	28 (36.4)	185 (36.7)	7 (21.9)	
>5 and <10 years	104 (17.0)	4 (5.2)	93 (18.4)	7 (21.9)	
≥10 years	143 (23.3)	19 (24.7)	120 (23.8)	4 (12.5)	
**SOC experience in the Outpatient Secondary Care Point model (from Portuguese, PASA)**					
No experience	486 (79.3)	59 (77.6)	412 (81.6)	15 (46.9)	**<0.001**
Up to 1 year	56 (9.1)	5 (6.6)	42 (8.3)	9 (28.1)	
>1 and ≤5 years	38 (6.2)	4 (5.3)	30 (5.9)	4 (12.5)	
>5 and <10 years	18 (2.9)	4 (5.3)	12 (2.4)	2 (6.2)	
≥10 years	15 (2.4)	4 (5.3)	9 (1.8)	2 (6.2)	

Note: PHC (Primary Health Care); SOC (Specialized Outpatient Care).

As can be seen, the majority reported having no previous experience as a preceptor or similar (59.6%) and working as a tutor for less than 1 year (58%). There was a relationship between the length of time as a tutor and the type of work (p-value = 0.012), so that the proportion of those who had been tutoring for less than 1 year seemed to be higher among tutors from PHC services (60.6%), while the proportion of those who had been working for more than 2 years was higher among state and regional tutors (41.3%) or tutors from SOC services (31.2%).

The proportion of tutors who reported more than 5 years’ experience in PHC was higher among tutors from PHC services (42.2%), compared to the other tutors (p-value < 0.001). With regard to working in SOC services in the Outpatient Secondary Care Point model (PASA, from Portuguese), approximately 80% of participants reported having no experience, with a lower proportion of these among those who worked as tutors in SOC services (46.9%) compared to the others (p-value < 0.001). The length of graduation completion (p-value < 0.001) and the higher level of education (p-value < 0.001) were also associated with the type of tutor, with a trend towards a longer length of finishing graduation and higher level of education among state and regional tutors or tutors in SOC services compared to PHC tutors.

Most of the participants worked as a tutor in the same workplace, especially in the case of PHC or SOC service tutors. With regard to protected weekly hours for tutoring, it was found that around 40% reported not having established hours, with a higher proportion among PHC tutors (45.8%), while the percentage of those who reported having more than two protected weekly hours was higher among SOC tutors (43.8%) (p-value < 0.001) (Table 2).

**Figure 2:**
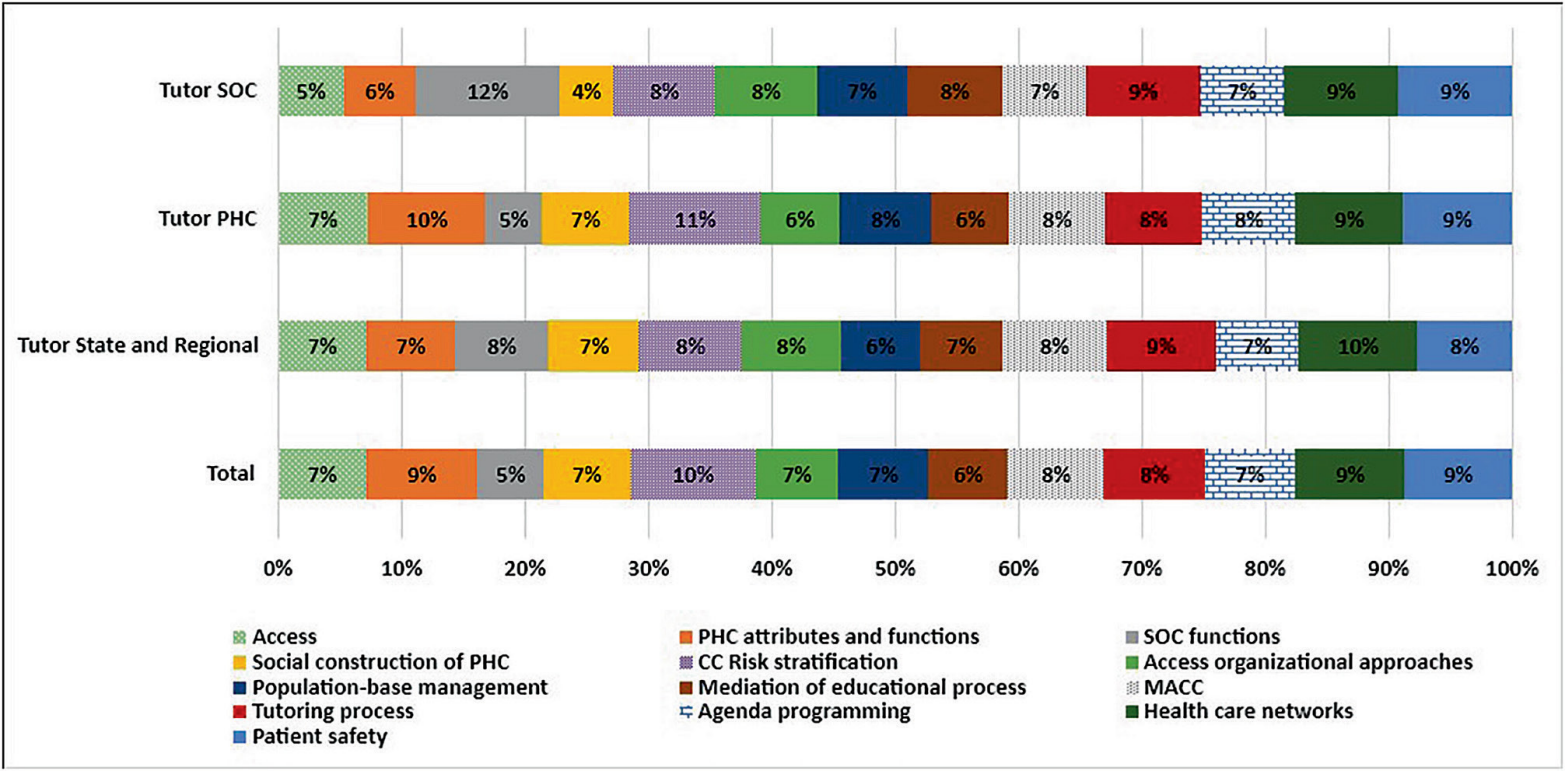
Topics considered important to be developed for PlanificaSUS tutors.

Notes: PHC (Primary Health Care); SOC (Specialized Outpatient Care); CC (chronic conditions); MACC (from Portuguese – Modelo de Atenção às Condições Crônicas – Model of Care for Chronic Conditions).

Figure 2 shows the percentages of positive responses to the topics considered relevant to working as a tutor, for all participants and by type of tutor. The questionnaire presented a list of 13 topics in a multiple-choice box format. Among SOC tutors, the most voted topic was SOC functions (12%) followed by tutoring process, healthcare networks and patient safety (9%). The topics least mentioned by SOC tutors were Social Construction of PHC (4%) and Access (5%). Among PHC tutors, the theme with the most votes was Risk Stratification of Chronic Conditions (11%), followed by Attributes and functions of PHC (10% of votes). The least popular themes among PHC tutors were SOC functions (5%), organizational approaches to access (6%) and mediation of educational processes (6%).

Among State and Regional tutors, the most voted topic was Health Care Networks (10%), followed by Tutoring Process (9%), while the topic least signaled as relevant was Population-based management (6%), followed by Access, Attributes and functions of PHC, Social construction of PHC, Mediation of educational processes and Agenda programming, all of which accounted for 7% of the votes.

In addition to the topics listed in the multiple-choice question, the questionnaire had an open question for the inclusion of other topics that might be of interest. There were 65 responses to this question with suggestions for new topics, which were categorized into the themes described in Table 3. The two suggestions that came up the most were “Management of Health Systems and Services” and the development of “Soft Skills Themes”, which include aspects of interpersonal relationships, attitudes and the individual’s ability to socialize.

**Table 3:** Other suggested topics to be developed for *PlanificaSUS* tutors – São Paulo, SP, Brazil, August-September, 2022.

Themes in category	N
Management of Health Systems and Services	13
Soft Skills Themes	11
Strategies for sharing care between professionals and points of care	7
Mental health in health services for care and health workers	7
Professional Ethics and Compliance in Health	5
Permanent Education and Continuing Education	4
Skills for planning and monitoring work processes	3
Health Surveillance	3
Urgent and Emergency Care	2
The role of the tutor	2
Digital Health	2
Territorialization	2
Spirituality and Health	1
Health Promotion Strategies	1
Integrative and Complementary Practices	1
Indigenous Health	1
Total	**65**

## DISCUSSION

Most of the participating tutors reported being cisgender women, of brown skin color, in the professional field of nursing, with a completed specialization, working as a tutor for less than a year, in the same workplace and in the Northeast region. Most did not report previous experience of working in outpatient clinics with the Outpatient Secondary Care Point model (from Portuguese, PASA) or as a preceptor or similar. Among those who reported previous experience with preceptorship, the following were mentioned: preceptorship of residency students, PET Health tutor or internship field teacher(s).

The characterization of the tutors taking part in the survey in terms of gender and race/color is similar to the way other groups of health professionals are presented in Brazil: cisgender and brown women. According to the World Health Organization (WHO) report on ‘The state of nursing in the world’, 89% of nursing professionals in the Americas are women^(17)^. It is noteworthy that despite appearing in a minority, there was representation of other races/colors in the group of HCP tutors. Women, particularly black women, are key players in maintaining essential services (such as health, education, social assistance, etc.) in emergency contexts^(18)^. On the front line, gender issues shape women’s experiences, considering that they are the majority in the sector and have historically carried out care work, strengthening the phenomenon known as “the feminization of healthcare”^(19)^.

Most of the tutors taking part in this study are from the professional field of nursing. This result shows that it is difficult for professionals from other areas to adhere to PHE actions^(20)^, including HCP. In this scenario, highlighting the need to focus on the importance of educational processes in the curricula of other health areas could result in the strengthening of interdisciplinarity and the adherence of other professional categories to PHE processes, such as those developed in the HCP.

A previous study investigated the training of nursing professionals in relation to their significant participation in educational processes, and found that these professionals play an important role as educators, highlighting the importance of seeking constant updating, as the job market increasingly demands critical and reflective professionals^(21)^. It is worth noting that, in the scenario of this study, HCP tutors were appointed or volunteered to take on this role, but did not receive additional remuneration for it. In this context, and given the evidence of the leading role played by nursing professionals in various PHE actions, the need for their official recognition in their work activities, as well as their remuneration, is highlighted.

Another important result of this study is that most of the participating tutors work in the Northeast. This finding is corroborated by the strong trajectory of this region in the development of Public Health programs, especially in PHC programs, observed since the 90s. In addition, it is worth highlighting the pioneering spirit of this region in PHE proposals, which first appeared in the Health Residency scenarios^(22)^. Given that a significant number of the health regions involved in *PlanificaSUS* are located in the Northeast, it was expected that tutors from these states would participate widely. The experiences accumulated in the Northeast allow us to think that strategies that encourage the involvement of health professionals in existing projects and spaces that use PHE can be an applicable and inexpensive alternative, and that this is a successful way of boosting the expansion of HCP.

With regard to the length of time tutors have been working under *PlanificaSUS*, there is a notable variation in the length of service of professionals working in PHC compared to those involved in SOC and management. This scenario is linked to the New Public Management model in PHC, which is characterized, among other things, by the introduction of Social Organizations, more flexible employment contracts and competition for resources in the public budget^(23)^. The phenomenon raises important questions about the fragility of employment relationships in the health sector, which tend to be more precarious among professionals hired to work in the Family Health Strategy^(23)^. This reality contributes to the instability of the workforce involved in PHE programs, pointing to the scarce prospect of professional growth and a sense of insecurity about the future. These conditions can, in turn, encourage workers to leave and constantly rotate, which is detrimental to the continuity and effectiveness of PHE actions.

On the other hand, among the tutors who represent the state health secretariats, whether at central or regional level, there is evidence of a longer time spent as *PlanificaSUS* tutors. One possible explanation is that the federal units, in management spaces, provide career civil servants with more stable employment relationships, thus contributing to the implementation and sustainability of SUS support projects such as *PlanificaSUS* ^(24)^.

Another important result is related to the tutor’s work setting. A significant percentage of tutors carry out *PlanificaSUS* activities in the same workplace as nurses, managers or other categories, in the case of PHC and SOC services. This configuration brings ambiguous perspectives, which can be understood as potential because the tutor is recognized as a member of the team and the service, and therefore tends to be legitimized by the other professionals because they are directly involved in organizing and qualifying the processes developed during the project. On the other hand, there is a risk of this professional being overloaded, especially when you look at the frequency with which nurses carry out care, management and tutoring duties, linked to learning aspects with the reference team.

In order to minimize this possible overload, weekly protected hours for the development of tutoring actions are recommended by the HCP methodology, in accordance with positive experiences in the organization of services and in professional praxis that have the dynamics of protected hours for PHE processes in their configuration^(25)^. However, around 40% of the tutors taking part in this study reported not having protected hours, especially in health centers. This result is corroborated by the national study on “Nursing Practices in the Context of PHC”, which shows that continuing education actions are the least prioritized compared to user care by appointment, spontaneous demand, team and service management activities, as well as health education actions^(26)^.

With regard to the previous experience with the Outpatient Secondary Care Point Model (from Portuguese, PASA), despite being the reference for organizing processes in the SOC services, through the HCP, this model presents itself as a new proposal for organizing specialized care^(12)^. This aspect was evidenced by the significant percentage of SOC tutors who reported having no experience in outpatient clinics based on this organizational model. In a context where the traditional model is dominant, secondary care service initiatives that adopt approaches in line with the principles of the secondary outpatient care point model (PASA) are rarely disseminated^(27)^. In this way, it is understood that *PlanificaSUS* is developing an active process of training actors to implement a model whose pillars ensure a break with the fragmented configuration of care provided to users in secondary care, regardless of the chronic condition established^(28)^.

When given the opportunity to indicate possible themes that they identify as relevant to their practice, the tutors present their needs in a heterogeneous way according to the context in which they work. The answers given by tutors from PHC centers are related to a deeper understanding of the clinic in order to organize the work process of the Family Health Strategy, while tutors from outpatient services state a greater need to understand how the secondary outpatient care point model (PASA) works and tutors linked to state management, whether at central or regional level, report a need for training related to the HCN.

In this sense, it is important to note that although everyone recognizes the need for PHE spaces, there is less demand for common themes. The diversity of interest in the topics can be explained by contextual variables, in which, on the one hand, PHC professionals are encouraged by the HCP methodology to qualify care for chronic conditions, and on the other, SOC professionals develop specialized care based on the logic of a model with different principles from those traditionally used in SUS outpatient units.

Of the themes that emerged from the open question, two categories stood out. The soft skills category stands out because it is a grouping of suggestions from participants that are aligned with the concept of social skills. It refers to behaviors that are valued for contributing to socially competent performance in interpersonal tasks. Emotion management and coping with stress; communication and interpersonal skills; decision-making and issue resolution; innovative thinking and critical analysis; self-knowledge and empathy are the five fundamental spheres of behavioral competencies that foster the development of individuals who are more proficient in dealing with everyday challenges and experiences^(29)^.

In addition, it can be understood that the activities and reflections offered by *PlanificaSUS* have consequences not only for the transformation of the work practices of the actors directly involved in HCP, but also for other health professionals and consequently in the qualification of care for the population. Valuing the meeting, listening and autonomy of workers and users is essential for PHE^(30)^. In this context, it is crucial to recognize that each professional, driven by their personal experiences and trajectories, develops approaches to face daily dilemmas. Continuous learning occurs through sharing experiences with each other, based on practice and interaction, and not limited to the mere transmission of knowledge.

The limitations of this study include the fact that the sample was intentional and not representative of all HCP tutors. However, the findings of this study show strengths in terms of characterizing the actors involved in processes that use PHE as a strategy for organizing work processes. Identifying the profile of tutors who develop the HCP in the context of *PlanificaSUS* provides potential for establishing development strategies for these strategic actors, as well as supporting future actions to recover the relationship between health services and continuing education processes. Another highlight is the fact that this is the first national study with primary data on the characteristics of health professionals who work as HCP tutors, covering all of the country’s geographical regions.

## CONCLUSION

This study made it possible to characterize the HCP tutor in the context of the *PlanificaSUS* project. It is worth noting that the tutor is one of the essential elements for the development of this methodology in the health regions, since they act as facilitators in the tutorial workshops, which involve the teams from the PHC units and SOC services. This allows them to reflect on the work process, directly supporting the teams in identifying what needs to be changed, based on the points made in relation to what they want to achieve and in planning improvement interventions, considering the local context and the needs of the people who use them.

Through this work, it was possible to present predominant characteristics among tutors, as well as differences according to tutor typologies, which can be considered by managers and institutions proposing actions involving the HCP methodology when it comes to selecting, choosing, and developing professionals who will act as HCP tutors. Future research should also focus on both the development aspects of these tutors and their evaluation of their role in the operationalization of the HCP.
